# Prospective study of the serum *Aspergillus*-specific IgG, IgA and IgM assays for chronic pulmonary aspergillosis diagnosis

**DOI:** 10.1186/s12879-019-4303-x

**Published:** 2019-08-06

**Authors:** Xiuqing Ma, Kaifei Wang, Xin Zhao, Yang Liu, Yanqin Li, Xiaotian Yu, Chunsun Li, David W. Denning, Lixin Xie

**Affiliations:** 1 Department of Pulmonary and Critical Care Medicine, Chinese PLA General Hospital, Beijing, China; 2 Academy for Life Science, Nankai University, Tianjin, China; 3 The National Aspergillosis Centre, Wythenshawe Hospital, The University of Manchester and Manchester Academic Health Science Centre, Manchester, UK

**Keywords:** Chronic pulmonary aspergillosis, *Aspergillus*-specific IgG antibody, *Aspergillus*-specific IgA antibody, *Aspergillus*-specific IgM antibody, Galactomannan

## Abstract

**Background:**

Chronic pulmonary aspergillosis (CPA) is an underdiagnosed and misdiagnosed disease and now increasingly recognised. However, the diagnosis of CPA remains challenging. In this study, we aimed to investigate the diagnostic values of serum *Aspergillus*-specific IgG, IgA and IgM antibodies in patients with CPA.

**Methods:**

The prospective study was performed at Chinese People’s Liberation Army General Hospital in Beijing, from January 2017 to December 2017. Adult patients with lung lesions presented as cavity, nodule, mass, bronchiectasis or severe fibrotic destruction with at least two lobes in CT imaging were enrolled. One hundred healthy persons were also enrolled as additional controls. The serum levels of *Aspergillus*-specific IgG, IgA and IgM antibodies and galactomannan (GM) levels were measured simultaneously by plate ELISA kit.

**Results:**

A total of 202 patients were enrolled in this study, including 42 CPA patients, 60 non-CPA patients and 100 healthy persons. The most common underlying lung diseases in CPA patients were bronchiectasis (28.6%) and COPD (19.0%). The most common symptoms in the CPA patients were cough (76.2%), sputum (71.4%), and fever (45.2%); chest pain (4.8%) was infrequent. Receiver operating characteristic (ROC) curve analysis revealed that the optimal CPA diagnostic cut-off of *Aspergillus*-specific IgG, IgA and IgM assays and GM test were 89.3 AU/mL, 8.2 U/mL, 73.3 AU/mL and 0.5μg/L, respectively. The serum levels of *Aspergillus*-specific IgG and IgA in CPA patients were higher than these in non-CPA patients or healthy persons. The sensitivities and specificities of *Aspergillus*-specific IgG, IgA, IgM tests and GM test were 78.6 and 94.4%, 64.3 and 89.4%, 50.0 and 53.7% and 71.4 and 58.1%, respectively.

**Conclusions:**

The sensitivity and specificity of serum *Aspergillus*-specific IgG assay are satisfactory for diagnosing CPA, while the performance of *Aspergillus*-specific IgA assay is moderate. *Aspergillus*-specific IgM assay and serum GM test have limited value for CPA diagnosis.

**Trial registration:**

NCT03027089. Registered 20 January 2017.

## Background

Chronic pulmonary aspergillosis (CPA) usually occurs in immunocompetent individuals with pre-existing chronic pulmonary disease, especially those leading to a structural lung damage such as pulmonary tuberculosis (PTB), chronic obstructive pulmonary disease (COPD), and bronchiectasis [1–4]. Hence CPA can be easily neglected or misdiagnosed due to its slow and insidious progression. It is estimated that there are 3 million patients with CPA worldwide and the 5-year survival rate of CPA is only 60% even when treated [5]. In China, the estimated annual CPA cases after PTB are more than 67,000, but most of the cases are undiagnosed due to lack of efficient diagnostic methods in routine practice [1]. Therefore, CPA has a high morbidity and mortality.

CPA diagnosis requires a combination of characteristic clinical and radiological findings together with demonstration of *Aspergillus* infection with growth of *Aspergillus* spp. on culture or the presence of elevated levels of *Aspergillus* antibody. Biopsy is most helpful in those with nodules but is unrewarding in cavitary disease as chronic inflammation and fibrosis is typically seen [6]. On the other hand, the clinical symptoms and radiological findings are often nonspecific due to the complexity of patients’ backgrounds, and the sensitivity of *Aspergillus* spp. culture of bronchoalveolar lavage fluid (BALF) or sputum is very poor [7]. The sensitivity and specificity of serum galactomannan *Aspergillus* antigen (GM) assay is at best only 43.3 and 80.8%, respectively [8]. Consequently, the diagnosis of CPA is challenging.

In 2016 the Infectious Diseases Society of America (IDSA) and separately the European Society for Clinical Microbiology and Infectious Diseases (ESCMID) in cooperation with the European Respiratory Society (ERS) published guidelines for the diagnosis of CPA [9, 10]. Both guidelines recommend measurement of *Aspergillus* IgG antibody as a key diagnostic step. However, the cut-off value for *A. fumigatus*-specific IgG remains to be established in China and varies for each assay format. Additionally, the diagnostic values of the other two *Aspergillus*-specific antibodies IgA and IgM remain unclear [11].

In this study we aimed to establish the optimal cut-off values of serum *Aspergillus*-specific IgG, IgA and IgM antibody assays and simultaneously investigated the diagnostic values of these three antibodies for CPA diagnosis.

## Methods

### Study design

This study, part of a prospective multicenter clinical trial (Clinical Trials. gov: NCT03027089), ran from January 2017 to December 2017 at Chinese People’s Liberation Army General Hospital, Beijing, China. All participants provided written informed consent and the study was approved by the ethics committee (S2017–015-01). Inclusion criteria were patients aged between 18 to 85 years, with lung lesions presenting as one or more cavities, nodules, masses, bronchiectasis or severe fibrotic destruction with at least two lobes of lung seen on computed tomography (CT) imaging. Exclusion criteria were as follows:

(1) Antifungal treatment for over 2 weeks within 3 months of enrollment (2) Incomplete records of clinical data or examination; (3) Pregnancy or lactation; (4) Severely immunocompromised patients, including a) recent history of neutropenia (<0.5 × 10^9^ neutrophils/L for>10 days); b) receipt of an allogeneic stem cell transplant, c) prolonged use of corticosteroids (excluding patients with allergic bronchopulmonary aspergillosis) at a mean minimum dose of 0.3 mg/kg/day of prednisone equivalent for >3 weeks, d) treatment with other recognized T cell immunosuppressants, such as cyclosporine, TNF-a blockers, specific monoclonal antibodies (such as alemtuzumab), or nucleoside analogues during the past 90 days, e) inherited severe immunodeficiency (such as chronic granulomatous disease or severe combined immunodeficiency) and f) acquired immunodeficiency syndrome (AIDS).

### Data and samples collection

During the first visit the demographic and clinical data of patients, including underlying diseases, chronic pulmonary or systemic symptoms, details of treatments and results of CT were recorded. Meanwhile, serum samples were collected and immediately refrigerated at 4 °C for *Aspergillus*-specific IgG antibody assay. The residual serum samples were frozen at − 80 °C for GM tests, *Aspergillus*-specific IgA and IgM antibody assays. C-reactive protein (CRP) tests, the peripheral total and differential white blood cell (WBC) counts, sputum direct microscopy for fungi and acid fast bacilli, and fungal, bacterial and mycobacterial culture were obtained. All patients underwent bronchoscopy and bronchoalveolar lavage fluid (BALF) and sometimes transbronchial biopsy specimens were obtained. The BAL procedure is according to ATS documents [12]. Normal saline is instilled through the bronchoscope, with a total volume that is between 100 and 300 ml and divided into three to five aliquots. In those with nodules or masses, a percutaneous aspirate was obtained, and direct microscopy and culture were undertaken within 5 days of hospitalisation. Control serum samples were collected from 100 healthy Chinese people with normal CT, CRP, and WBC count. All serum samples were immediately refrigerated at 4 °C for *Aspergillus*-specific IgG assay. The residual serum samples were also frozen at − 80 °C for *Aspergillus*-specific IgA and IgM assays and GM test.

### Definition of CPA diagnosis

CPA diagnosis was determined according to revised ESCMID/ERS CPA diagnosis criteria in which serum *Aspergillus*-specific IgG or serum GM assays were not considered as microbiological evidence. Diagnosis of CPA required the following criteria: 1) relevant symptoms have been present for at least 3 months (sputum, cough, hemoptysis, fever, chest pain, and/or shortness of breath); 2) the presence of a pulmonary lesion with evidence of cavitation, fungal ball, pleural thickening and/or upper lobe fibrosis; 3) microbiological evidence of *Aspergillus* infection: direct microscopy for hyphae from biopsy or resection of lung tissue, *Aspergillus* culture growth or repeated positive GM test from BALF, repeated *Aspergillus* growth from sputum culture, and 4) the exclusion of other pulmonary diseases associated with similar disease presentation (e.g., mycobacteria, coccidioidomycosis, and lung cancer) [10]. The diagnosis of CPA in each patient was confirmed by at least one experienced clinician.

### Serum Aspergillus-specific IgG, IgA, IgM and GM assays

Serum *Aspergillus*-specific IgG, IgM antibodies and GM were measured by using enzyme linked immunosorbent assay (ELISA; Dynamiker, Tianjing, China). Serum *Aspergillus*-specific IgA levels were measured in batches by using ELISA (IBL, Hamburg, Germany). Each assay was performed according to the manufacturers’ instructions with manual pipetting and washing. Optical density (OD) was measured with a microplate reader in 96-cell plate (BioTek Instruments, Highland Park, USA).

### Statistical analysis

Statistical analyses were performed using SPSS (version 22, IBM, NY, USA). The data were expressed as numbers and percentages for categorical variables and as means and standard deviations (mean ± SDs) for quantitative variables. Receiver operating characteristics (ROC) analysis was performed for *Aspergillus*-specific IgG assay. Area under the ROC curve (AUC) was shown with 95% Wilson confidence intervals (CI). Optimal diagnostic cut-off value for *Aspergillus*-specific IgG were calculated using Youden’s J statistic (sensitivity+specificity-1). Normality test was applied to evaluate all quantitative variables to select the appropriate test. Comparisons between CPA group, non-CPA group, and healthy controls were done using the *t*-test. The McNemar χ2 test and kappa test were used to compare sensitivity, specificity, positive likelihood ratio (PLR), and negative likelihood ratio (NLR) between the assays. In all analyses, *P* < 0.05 was considered significant.

## Results

### Patient characteristics

**Fig. 1:**
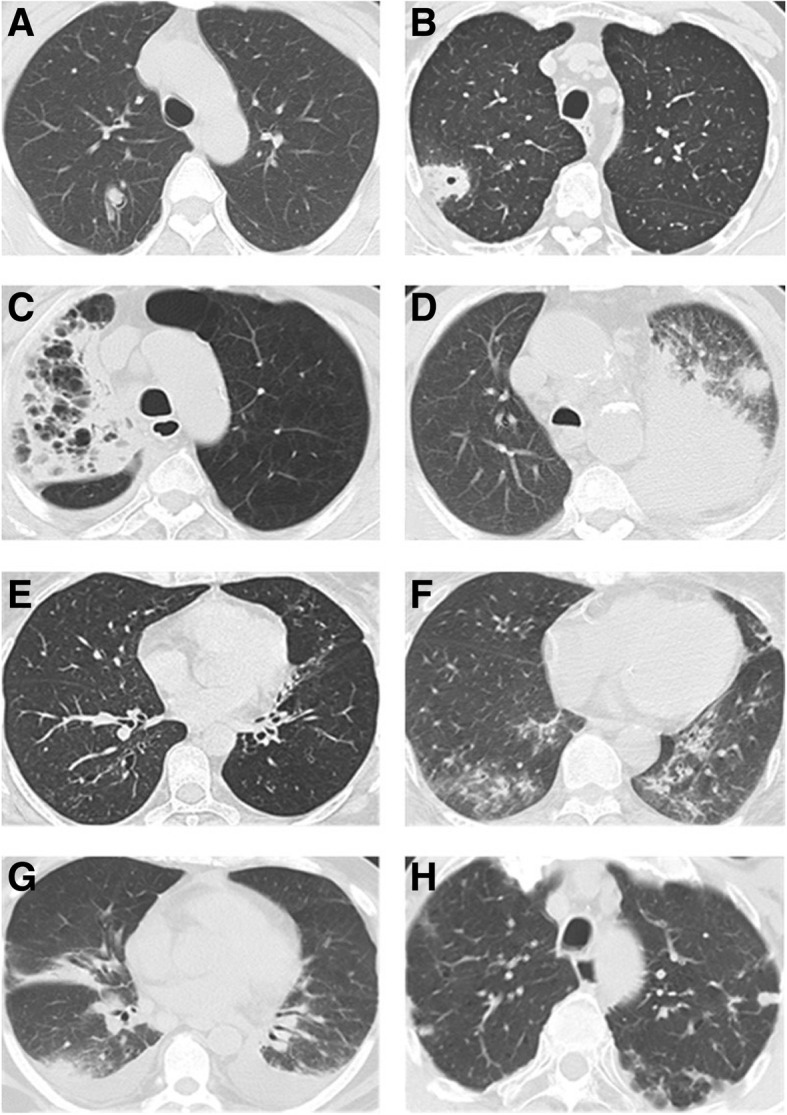
Representative examples of CT appearances in patients with CPA. CT images show: **a** a small fungus ball with an air crescent sign in the right upper lobe (**b**) aspergillus nodule with cavitary lesions and halo sign in the right upper lobe, **c** reticular pattern of inflammatory or fibrotic change surround small lung bullae and areas of consolidation and some pleural thickening and indrawing of fat in the right upper lobe, **d** consolidaton, with multiple nodules and marked loss of volume in the left lung, **e** bronchiectasis in the right upper and lower lobe and left lower lobe, **f** bronchiectasis and inflammatory infiltrates in both lower lobes, **g** consolidation in the right middle lobe, irregular nodule with surrounding ground glass in the right lower lobe and bilateral pleural effusions, **h** aspergillus nodules in both lower lobes and reticular pattern in the left upper lobe

**Fig. 2:**
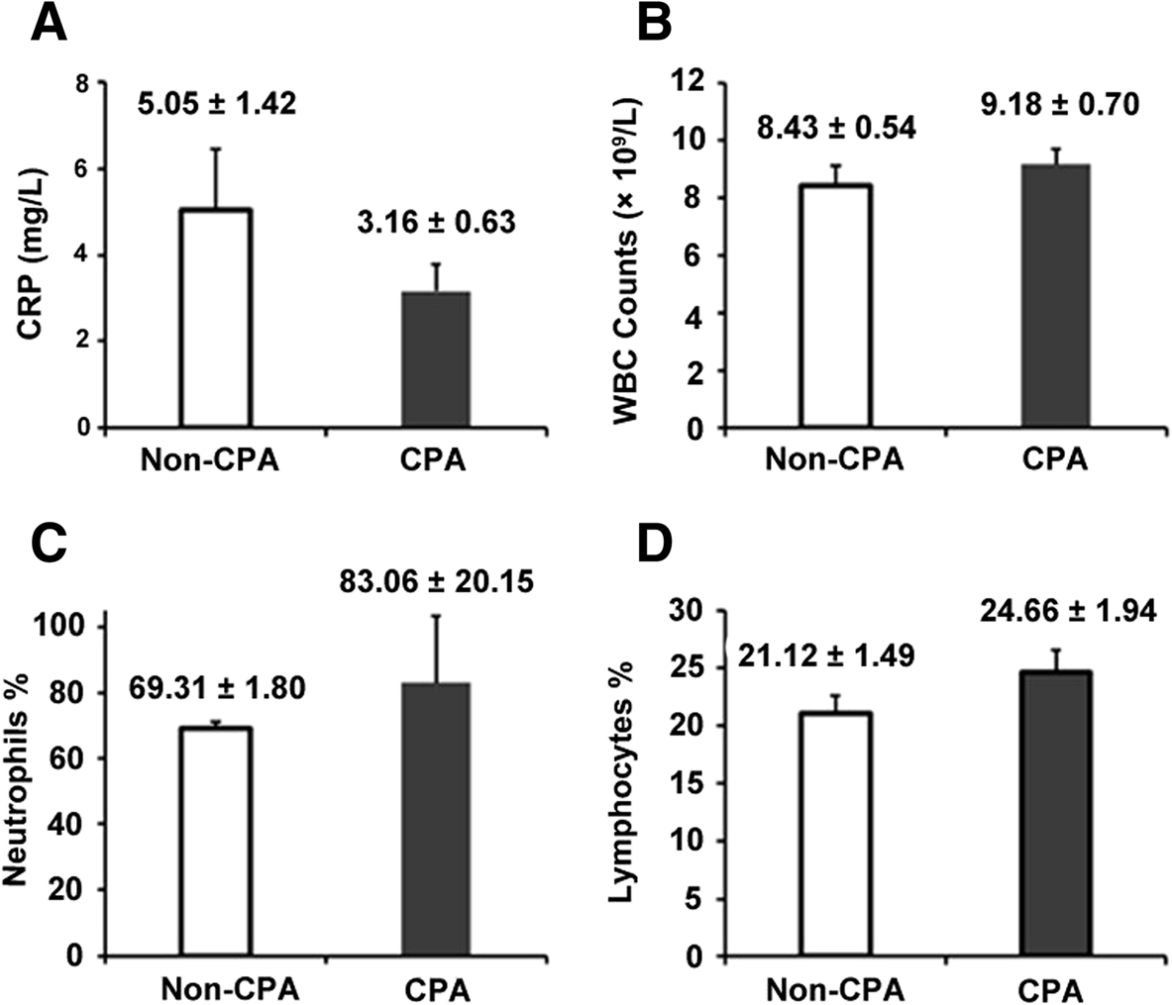
Comparisons of CRP concentration, WBC count, neutrophil percentage, and lymphocyte percentage in peripheral blood between CPA and non-CPA patients. The *p*-Values were: **a**, 0.229; **b**, 0.398; **c**, 0.500; **d**, 0.151, respectively. Mean and standard error are shown

Between January 2017 to December 2017, 128 patients and 100 healthy persons were recruited. A total of 26 patients were excluded from this study due to the following reasons: for 7 patients we did not have complete clinical records or laboratory data because of dropout or death before diagnosis; 8 patients received antifungal therapy for over 2 weeks within 3 months of enrollment; 11 patients had severe immunodeficiency due to allogeneic stem cell transplant or T cell immunosuppressants. Eventually 102 patients were included and 42 patients were diagnosed with CPA (41.2%). The pathogen was identified from sputum (*n* = 15, 35.7%), BALF culture (*n* = 6, 14.3%), lung resection surgery (*n* = 4, 9.5%), bronchoscopy biopsy (*n* = 2, 4.8%), percutaneous lung biopsy (*n* = 2, 4.8%) and BALF GM assay (*n* = 13, 31.0%). Among 21 culture positive cases, 17 cases were identified *as Aspergillus fumigatus*, 3 were identified *as Aspergillus niger*, one was identified *as Aspergillus flavus*. The most common underlying lung diseases in CPA patients were bronchiectasis (28.6%) and COPD (19.0%). The most common symptoms in the CPA patients were cough (76.2%), sputum (71.4%), and fever (45.2%). Chest pain (4.8%) was not the main symptom of these CPA patients in our study (Table 1). Representative chest CT findings from the index cases are shown in Fig. 1. Healthy controls included 54 male and 46 female and the mean age was 43.0 ± 9.7 years (mean ± SD). There were no statistical differences in CRP concentration, WBC count, neutrophil percentage, and lymphocyte percentage of peripheral blood between CPA group and non-CPA group, *P* > 0.05 (Fig. 2).

**Table 1:** CPA and Non-CPA patient characteristics

Characteristic	CPA patients (*n* = 42)	Non-CPA patients (*n* = 60)
Male gender	20 (47.6%)	37 (61.7%)
Age (years) (mean ± SD)	54.6 ± 16.8	52.7 ± 18.1
Underlying lung diseases		
Bronchiectasis	12 (28.6%)	18 (30.0%)
COPD	8 (19.0%)	10 (16.7%)
Emphysema	6 (14.3%)	1 (1.7%)
Previous tuberculosis	3 (7.1%)	4 (6.7%)
Interstitial pneumonia	2 (4.8%)	2 (3.3%)
Asthma	2 (4.8%)	8 (13.3%)
Pulmonary embolism	2 (4.8%)	0
Prior pneumonia	2 (4.8%)	5 (8.3%)
Chronic bronchitis	2 (4.8%)	8 (13.3%)
Pulmonary sarcoidosis	1 (2.4%)	2 (3.3%)
Eosinophilic pneumonia	1 (2.4%)	0
No underlying pulmonary diseases	4 (9.5%)	5 (8.3%)
Underlying systemic diseases		
Diabetes mellitus	6 (14.3%)	9 (15.0%)
Heart failure	5 (11.9%)	8 (13.3%)
Rheumatic disease	5 (11.9%)	3 (5.0%)
Chronic renal failure	3 (7.1%)	4 (6.7%)
Chronic pulmonary or systemic symptoms		
Cough	32 (76.2%)	45 (75.0%)
Sputum	30 (71.4%)	38 (63.3%)
Fever	19 (45.2%)	34 (56.7%)
Dyspnea	17 (40.5%)	30 (50.0%)
Hemoptysis	11 (26.2%)	9 (15.0%)
Chest pain	2 (4.8%)	2 (3.3%)
Chest CT findings		
Bronchiectasis	12 (28.6%)	18 (30.0%)
Consolidation	8 (19.0%)	9 (15.0%)
Cavitation	5 (11.9%)	5 (8.3%)
Pleural effusion	4 (9.5%)	8 (13.3%)
Nodules	4 (9.5%)	19 (31.7%)
Bullae of lung	3 (7.1%)	1 (1.7%)
Fibrotic changes	3 (7.1%)	5 (8.3%)
Pleural thickening	3 (7.1%)	3 (5.0%)
Fungal ball	2 (4.8%)	0
Mass	2 (4.8%)	6 (10.0%)
Halo sign	2 (4.8%)	0
Air crescent sign	1 (2.4%)	0

*COPD* chronic obstructive pulmonary disease, *CPA* chronic pulmonary aspergillosis, *CT* computed tomography

### Determination of the cut-off point

**Fig. 3:**
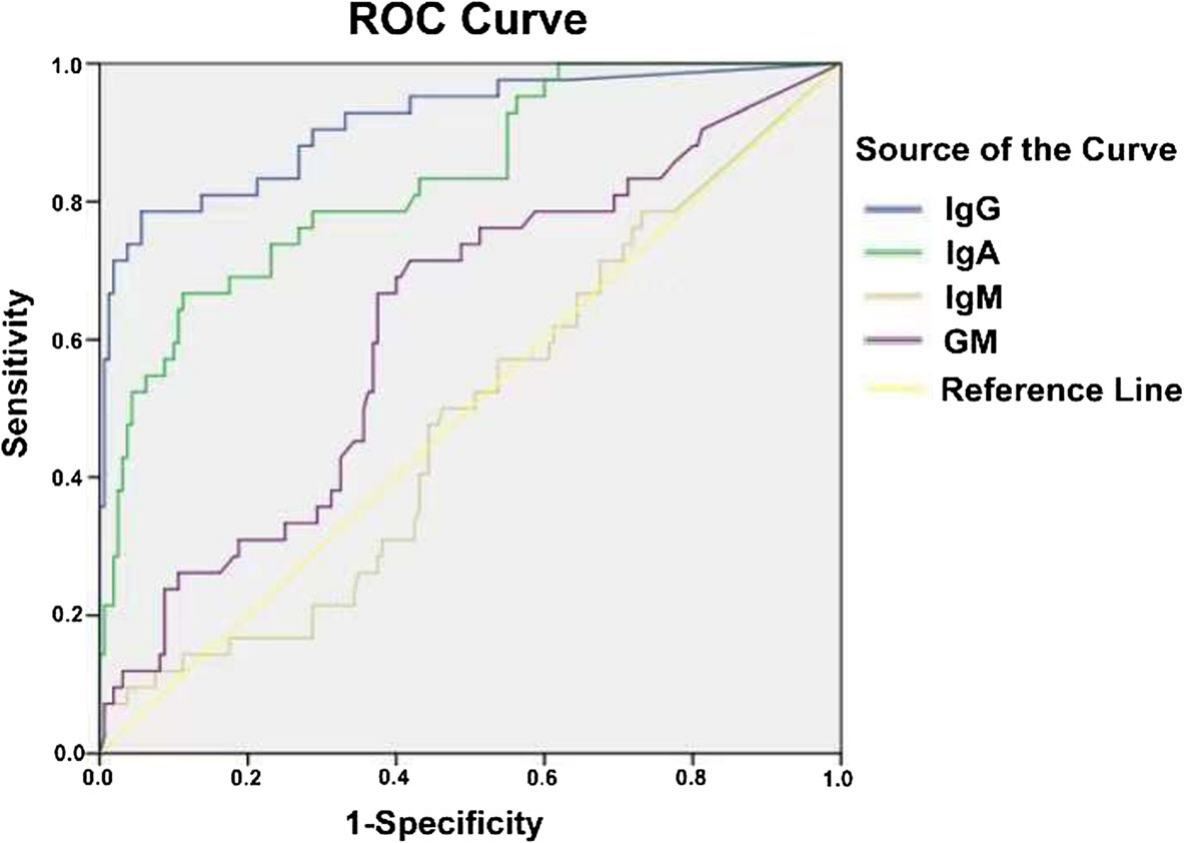
ROC analysis for CPA patients, non-CPA patients and healthy controls. The AUC of *Aspergillus* IgG, IgA, IgM assays and GM test were 0.915 (95% CI, 0.860 to 0.969), 0.833(95% CI, 0.764 to 0.903), 0.488 (95% CI, 0.391to 0.584), 0.622 (95% CI, 0.527 to 0.716) respectively

ROC analysis indicated that the AUC of *Aspergillus* IgG assay was highest of the 3 assays (0.915), then *Aspergillus* IgA assay (0.833) (Fig. 3). The optimal cut-off value of *Aspergillus* IgG, and IgA assays for diagnosing CPA were 89.3 AU/mL and 8.2 U/mL, respectively (Table 2).

**Table 2:** Performance of potential diagnostic cut-offs

Assay (Unit)	Diagnostic cut-off	Sensitivity	Specificity	Youden’s J statistic
IgG (AU/mL)	70.3	81.0%	83.1%	0.641
	82.7	78.6%	91.2%	0.698
	89.3	78.6%	94.4%	0.729
	91.8	76.2%	94.4%	0.706
	118.6	71.4%	96.9%	0.683
IgA (U/mL)	6.1	71.4%	78.1%	0.496
	7.2	69.0%	82.5%	0.515
	8.2	64.3%	89.4%	0.537
	9.8	57.1%	93.7%	0.509
	10.7	52.4	95.0%	0.474
IgM (AU/mL)	53.3	61.9%	38.7%	0.007
	66.5	52.4%	48.1%	0.005
	73.3	50.0%	53.7%	0.038
	81.9	45.2%	57.5%	0.027
	84.0	42.9%	59.4%	0.022
GM (ug/L)	0.40	76.2%	45.6%	0.218
	0.45	73.8%	51.2%	0.251
	0.50	71.4%	58.1%	0.296
	0.60	45.2%	65.0%	0.102
	0.70	33.3%	73.1%	0.065

### Clinical value of IgG, IgM, IgA and GM assay

**Fig. 4:**
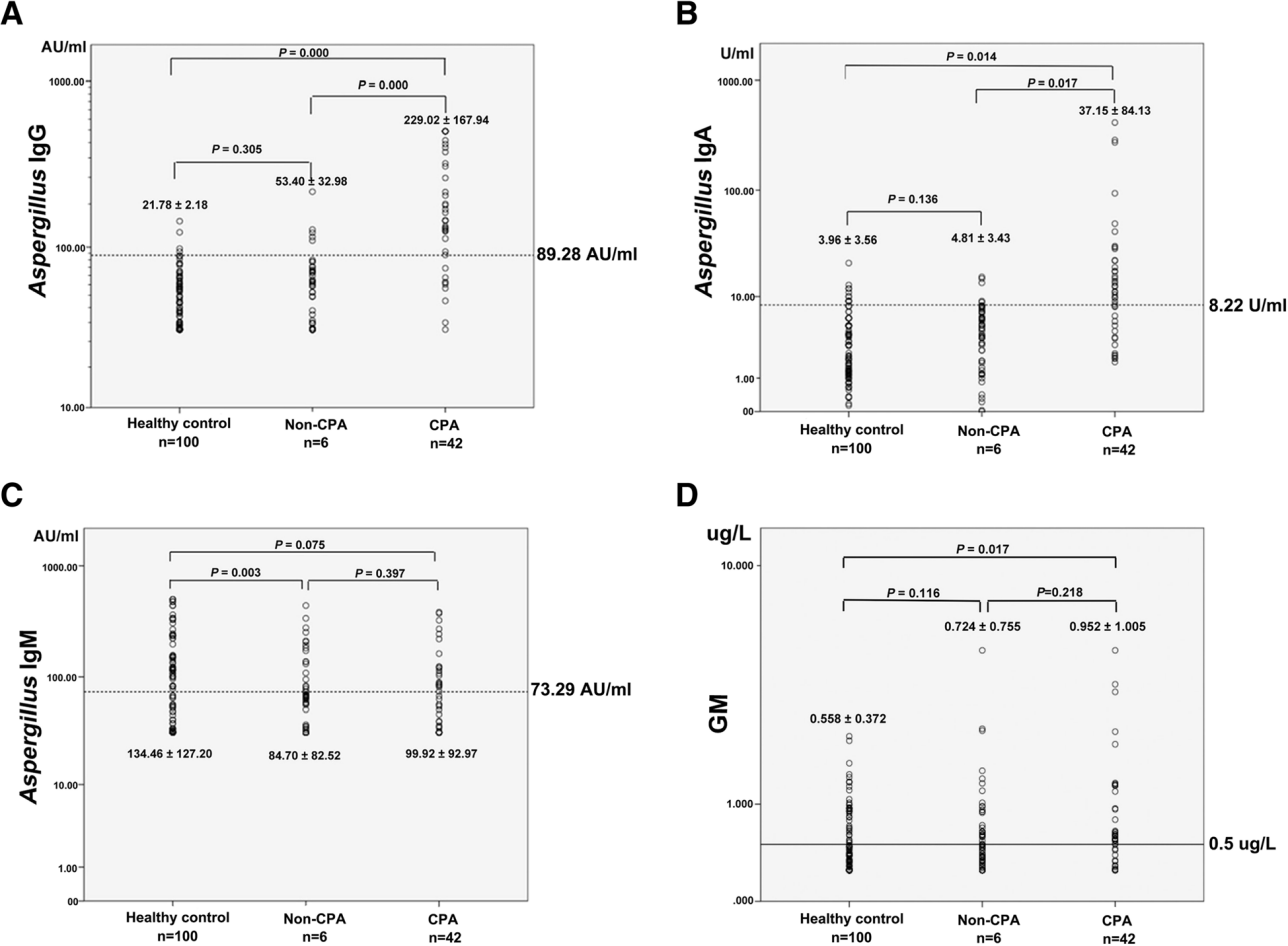
Average *Aspergillus* IgG (**a**), IgA (**b**) and IgM (**c**) level and GM (**d**) in serum from healthy control group, non-CPA group and CPA group. Data represent the means±SDs

Using new cut-off levels, *Aspergillus* IgG assay had the highest sensitivity (78.6%) and specificity (94.4%). The sensitivity and specificity of *Aspergillus* IgA assay, *Aspergillus* IgM assay and GM test were 64.3 and 89.4%, 50.0 and 53.7%, 71.4 and 58.1%, respectively (Table 3). Using the mean value, the *Aspergillus* IgG level of CPA patients was significantly higher than that of non-CPA patients or healthy controls (*P* < 0.001), but there was no statistical difference between non-CPA patients and healthy controls, *P* = 0.305 (Fig. 4).

**Table 3:** The CPA detection rates of each assay compared with the CPA diagnosis rate of gold standard

	Cut off value	Sensitivity(95% CI)	Specificity(95% CI)	PLR(95% CI)	NLP(95% CI)	Kappa index	*P* value
*Aspergillus* IgG test	89.3 AU/mL^a^	78.6% (62.8–89.2)	94.4% (89.3–97.2)	14.0 (7.3–26.9)	0.23 (0.13–0.41)	0.729	0.000
	80.0 AU/mL^b^	78.6% (62.8–89.2)	89.4% (83.3–93.5)	7.4 (4.6–11.9)	0.24 (0.13–0.43)	0.635	0.000
	120.0 AU/mL^c^	71.4% (55.2–83.8)	96.9% (92.5–98.8)	22.9 (9.4–55.3)	0.29 (0.18–0.48)	0.728	0.000
*Aspergillus* IgA test	8.2 U/mL^a^	64.3% (48.0–78.0)	89.4% (83.3–93.5)	6.1 (3.7–10.0)	0.40 (0.27–0.60)	0.527	0.000
	8.0 U/mL^b^	66.7% (50.4–80.0)	84.4% (77.6–89.4)	4.3 (2.8–6.5)	0.39 (0.26–0.61)	0.465	0.000
	12.0 U/mL^c^	47.6% (32.3–63.4)	95.6% (90.8–98.1)	10.9 (4.9–24.0)	0.55 (0.41–0.73)	0.498	0.000
*Aspergillus* IgM test	73.3 AU/mL^a^	50.0% (34.4–65.6)	53.7% (45.7–61.6)	1.1 (0.8–1.5)	0.93 (0.7–1.3)	0.026	0.665
	80.0 AU/mL^b^	47.6% (32.3–63.4)	55.6% (47.6–63.4)	1.1 (0.8–1.5)	0.94 (0.7–1.3)	0.017	0.782
	120.0 AU/mL^c^	21.4% (10.8–37.2)	68.8% (60.9–75.7)	0.7 (0.4–1.3)	1.14 (1.0–1.3)	0.085	0.213
GM test	0.50 μg/L^a^	71.4% (55.2–83.8)	58.1% (50.1–65.8)	1.7 (1.3–2.2)	0.49 (0.3–0.8)	0.199	0.001
	0.65μg/L^b^	35.7% (22.0–52.0)	70.6% (62.8–77.4)	1.2 (0.8–1.9)	0.91 (0.7–1.1)	0.054	0.428
	0.85μg/L^c^	31.0% (18.1–47.2)	76.3% (68.8–82.5)	1.3 (0.8–2.2)	0.91 (0.7–1.1)	0.013	0.848
*Aspergillus* IgG + IgA test^d^		83.3% (68.0–92.5)	87.5% (81.1–92.0)	6.7 (4.3–10.3)	0.19 (0.1–0.4)	0.636	0.000

^a^Cut off value form this study

^b^lower detection limit of the kit

^c^upper detection limit of the kit

^d^combined detection of *Aspergillus* IgG and IgA

## Discussion

To our best of knowledge, this is the first prospective study to simultaneously investigate the clinical value of *Aspergillus*-specific IgG, IgA and IgM in diagnosis of CPA. Although *Aspergillus*-specific IgG were widely used in the diagnosis of CPA, most studies were retrospective and a few studies have reported the cut-off value. Only one study reported the *Aspergillus*-specific IgG cut-off value of Dynamiker kit [13]. The results of this study suggest that *Aspergillus*-specific IgG assay (cut-off of 89.3 AU/mL) has the highest sensitivity and specificity among three *Aspergillus*-specific antibodies assays, followed by *Aspergillus*-specific IgA assay. GM test has limited value in the diagnosis of CPA and *Aspergillus*-specific IgM assay has no value at all.

The size of the problem of CPA worldwide has only recently been appreciated. The positive rate of CPA in this patient cohort was 41.2%. That might be the enrolled patients were high risk for CPA, or the number of CPA patients in China was larger than the estimated data. Additionally, due to humid weather, the incidence rate of CPA might be higher in warm and damp regions than dry and cold regions, like south China compared with north China. Therefore, it was urgent to evaluate the performance of serum *Aspergillus* specific antibodies assays which can be used for diagnosis of CPA.

The management policy in China for patients with possible or confirmed pulmonary tuberculosis requires transfer to a specialized isolation hospital. As a result, we only enrolled 3 patients in our study with a history of PTB. Fortunately, two tuberculosis hospitals were included in our ongoing multicenter program. After excluding PTB and nontuberculous mycobacterial (NTM), according to our data, the most common underlying disease of CPA patients was bronchiectasis, followed by COPD. However, some studies reported that COPD was considered as the primary underlying diseases [6, 14, 15]. Possible factors contributing to the difference are as follows. Firstly, 30–60% of COPD patients accompanied bronchiectasis [16–18]. It was not clear whether COPD patients in the above reports have bronchiectasis. Furthermore, of the patients with COPD-bronchiectasis overlap, 23.5% had a positive culture for *A. fumigatus* versus 10.5% of COPD patients without bronchiectasis [19]. Hence COPD-bronchiectasis overlap might be separate analysis. Secondly, in the treatment of bronchiectasis, the abuse of antibiotics might be related to the increase of *Aspergillus* infection. Thirdly, aetiologies varied considerably among different geographic regions [20]. Fourthly, the prevalence of bronchiectasis in China might be higher compared with western countries. Finally, the number of CPA patients which we enrolled was relatively small.

The main systemic symptoms were cough, sputum production and fever, but the rate of chest pain was not high. The rate of haemoptysis (26.2%) was lower than other reports (49 and 63.8%) [6, 15]. One of the possible reasons is that the numbers of bronchiectasis and PTB, which contribute to haemoptysis, were small in our study. Nineteen of 42 CPA patients (including 17 inpatients and 2 outpatients) had pyrexia during the study period. The rate of fever (45.2%) was higher than other published papers reported (17.4 and 21%) [6, 15]. Among this group of CPA patients, 13 patients had other infections, 3 patients have connective tissue disease and 4 patients had pyrexia probably attributable to CPA. In our CPA patients, specific chest CT findings, like halo sign and air crescent sign, were rarely seen. The rate of pulmonary cavitation (11.90%) is much lower than reported (26, 94.2%) [15, 21]. These 42 CPA patients come from our hospital and only 3 patients have previous tuberculosis. That may be the reason for the low rate of pulmonary cavitation.

We compared four serum inflammation markers, CRP concentration, WBC count, neutrophil percentage and lymphocyte percentage between patients with CPA and without CPA, but there was no statistical difference in two groups. It showed that inflammation markers could not be used in diagnosing CPA [22, 23]. The sensitivity of *Aspergillus* culture from the respiratory tract specimens was 50.0% (21/42) in our study. *Aspergillus* culture had definite limitations, including poor sensitivity, potential contamination and labor intensity.

Serum *Aspergillus* IgG antibody assay performed well in terms of ROC AUC. At cut-off of 89.3 AU/mL, the sensitivity, specificity and Youden index from CPA group, non-CPA group and healthy controls were 78.6, 96.0% and 0.746, respectively. Similarly, an investigation with the kits from the same manufacturer reported that the sensitivity, specificity and Youden index were 77, 97% and 0.74, respectively, at a cut-off of 65 AU/mL [13]. Except for the difference in control populations, the difference in reported optimal cut-off values was mainly because of the upgrade of kits. In our study we used the newest version of the *Aspergillus*-specific IgG assay kit from Dynamiker, which is approved by the China State Food and Drug Administration. The new manufacturer’s recommended cut off is 80–120 AU/mL, which is different from the one used in study from Page et al. (50–60 AU/mL). Noticeably, Dynamiker *Aspergillus*-specific IgG antibody assay uses purified galactomannan as its sole antigen, while other kits use fungi extract or recombinant antigen [11]. The ROC AUC performance of the assay was proved reasonable and equivalent to other available commercial ELISA assays [13]. The serum *Aspergillus* IgG antibody assay are well accepted for diagnosis of CPA in developed countries, such as Japan, UK, France [24–26]. Consequently, it was important to evaluate the performance of serum *Aspergillus* IgG antibody assay in other countries, like our country China, in order to encounter the raising CPA issues.

We also evaluated the clinical value of serum *Aspergillus*-specific IgA antibody, *Aspergillus*-specific IgM antibody and GM assays in CPA diagnosis. Positive *Aspergillus* IgA antibody were found in up to 76% of CPA cases, which was higher than that from our study (57.1%) [27, 28]. The difference might be due to our small sample pool and selection bias and also be due to the anti-IgA ELISA test performances by itself. In this CPA group, 2 out of 42 patients had positive *Aspergillus* IgA antibody and negative *Aspergillus* IgG antibody. However, combined detection did not have any obvious advantage. IgA antibody was normally associated with mucosal immunity, thus BALF *Aspergillus* IgA antibody might worth to be considered for diagnosis of CPA. Consistently with current literatures, the sensitivity and specificity of the *Aspergillus* IgM antibody test and GM test were not satisfied, although the reported positive *Aspergillus* IgM antibody level was reported in up to 50% of CPA cases [11, 27, 28], which was higher than our study. IgM was the earliest antibody in the immune response and typically associated with the acute phase of an infection. Therefore, *Aspergillus*-specific IgM antibody might be useful for diagnosis of sub-acute invasive pulmonary aspergillosis. More prospective controlled studies were needed to evaluate the clinical value of IgA and IgM antibody assays in CPA diagnosis. According to the new guideline published by IDSA, BALF but not serum GM should be used in diagnosis of CPA [9, 10]. However, the cut-off value of BALF GM test was still controversial [9, 29, 30].

There are two limitations in our current study. First, we did not investigate the relationship between the *Aspergillus*-specific antibody levels and antifungal therapy. ERS stated that *Aspergillus*-specific IgG will decrease upon successful treatment [10], but research on *Aspergillus*-specific IgA and IgM was rare. Second, the analysis of the *Aspergillus*-specific antibody levels in different subtypes of CPA is lacking. To date there are no studies in this field.

## Conclusions

In conclusion, the Dynamiker *Aspergillus*-specific IgG test at a cut-off value of 89.3AU/mL has a high sensitivity and specificity in a Chinese population suffering of pulmonary disease. It performs best compared with *Aspergillus*-specific IgA, IgM assays and GM test and should be used in preference. The performance of *Aspergillus*-specific IgA assay is moderate. *Aspergillus*-specific IgM assay and serum GM test have limited value should not recommended for CPA diagnosis.

## Data Availability

The datasets used and/or analysed during the current study are available from the corresponding author on reasonable request.

## Abbreviations

AUC: Area under the curve
BALF: Bronchoalveolar lavage fluid
COPD: Chronic obstructive pulmonary disease
CPA: Chronic pulmonary aspergillosis
CPR: C-reactive protein
CT: Computed tomography
ELISA: Enzyme linked immunosorbent assay
ERS: European Respiratory Society
ESCMID: European Society for Clinical Microbiology and Infectious Diseases
GM: Galactomannan
IDSA: Infectious Diseases Society of America
IgA: Immunoglobulin A
IgG: Immunoglobulin G
IgM: Immunoglobulin M
NLR: Negative likelihood ratio
NPV: Negative predictive value
NTM: Nontuberculous mycobacterial
OD: Optical density
PLR: Positive likelihood ratio
PPV: Positive predictive value
PTB: Pulmonary tuberculosis
ROC: Receiver operating characteristics
WBC: White blood cell
